# A Highly Efficient Heterogeneous Processor for SAR Imaging

**DOI:** 10.3390/s19153409

**Published:** 2019-08-03

**Authors:** Shiyu Wang, Shengbing Zhang, Xiaoping Huang, Jianfeng An, Libo Chang

**Affiliations:** School of Computer Science and Engineering, Northwestern Polytechnical University, Xi’an 710072, China

**Keywords:** heterogeneous array, SAR imaging, data cross-placement, computing resource management

## Abstract

The expansion and improvement of synthetic aperture radar (SAR) technology have greatly enhanced its practicality. SAR imaging requires real-time processing with limited power consumption for large input images. Designing a specific heterogeneous array processor is an effective approach to meet the power consumption constraints and real-time processing requirements of an application system. In this paper, taking a commonly used algorithm for SAR imaging—the chirp scaling algorithm (CSA)—as an example, the characteristics of each calculation stage in the SAR imaging process is analyzed, and the data flow model of SAR imaging is extracted. A heterogeneous array architecture for SAR imaging that effectively supports Fast Fourier Transformation/Inverse Fast Fourier Transform (FFT/IFFT) and phase compensation operations is proposed. First, a heterogeneous array architecture consisting of fixed-point PE units and floating-point FPE units, which are respectively proposed for the FFT/IFFT and phase compensation operations, increasing energy efficiency by 50% compared with the architecture using floating-point units. Second, data cross-placement and simultaneous access strategies are proposed to support the intra-block parallel processing of SAR block imaging, achieving up to 115.2 GOPS throughput. Third, a resource management strategy for heterogeneous computing arrays is designed, which supports the pipeline processing of FFT/IFFT and phase compensation operation, improving PE utilization by a factor of 1.82 and increasing energy efficiency by a factor of 1.5. Implemented in 65-nm technology, the experimental results show that the processor can achieve energy efficiency of up to 254 GOPS/W. The imaging fidelity and accuracy of the proposed processor were verified by evaluating the image quality of the actual scene.

## 1. Introduction

Aerospace synthetic aperture radar (SAR) can be all-time and all-weather to obtain high-precision microwave images and other value-added products over large areas, and it has an extensive range of applications in remote sensing, environmental monitoring, geographical mapping, war zone surveillance, precision guidance, and reconnaissance [1,2,3,4].

Extensions and modifications of the SAR technology have significantly increased its practicality and applications. The demand for high-resolution and wide-swath (HRWS) SAR imaging is growing, especially in the areas of ocean observation, geological survey, and environmental protection. In 1978, the United States launched the first spaceborne SAR named Seasat-1. It is a satellite specifically designed for telemetry of the Earth’s oceans, and is aimed at realizing the possibility of global satellite monitoring of the oceans and determining the system requirements for marine remote sensing satellites. RADARSAT-1 was successfully launched in Canada in 1995 [5]. It not only provided Canada with a large amount of all-weather and all-time SAR data, but also provided useful information for commercial and scientific users in disaster management, agriculture, mapping, hydrology, forestry, oceanography, ice research, and coastal monitoring. In January 2006, Japan launched the Advanced Land Observing Satellite (ALOS) [6]. The Phased Array type L-band Synthetic Aperture Radar (PALSAR) that it carried was an L-band SAR sensor that is not affected by atmospheric conditions, cloud cover, and other related conditions, so it can be used for ground observations around the clock. In June 2007, the Terra SAR-X was launched by the German National Space Center. Its X-band SAR radar reliably provided high-resolution weather conditions and wide-area radar images with superior geometric accuracy over any other spaceborne SAR sensor [7]. Moreover, for both civil and military applications, it is desired to monitor moving targets, including ground moving target indication/ground moving target imaging (GMTI/GMTIm) [8,9].

For SAR processing systems, SAR imaging time accounts for most of the processing time, and directly brings a significant impact on system throughput and rapid response capability. The imaging delay of SAR will seriously affect the subsequent image processing, such as content analysis, risk diagnosis, and feature extraction. SAR imaging efficiency plays a very important role in the SAR system platform, and it can directly affect the throughput and rapid response capability of the entire platform.

Spaceborne and airborne real-time SAR imaging is the most direct and effective real-time imaging implementation approach, which can quickly provide SAR image data for SAR applications while significantly reducing the communication burden of air-to-ground data links [10]. At the same time, the working environment of spaceborne and airborne imaging systems is harsh, and the power consumption of the processor is also severely limited. Therefore, real-time and low power consumption are two essential items that must be met by spaceborne/airborne SAR imaging processors.

Since SAR imaging requires a large amount of two-dimensional parallel computing, it is difficult for a single multi-core central processing unit (CPU) to meet its real-time requirements. The SAR imaging scheme with multiple CPU nodes has high power consumption and low processing efficiency, and cannot be applied in spaceborne/airborne SAR processing. Generally, heterogeneous schemes such as CPU + GPU, CPU + DSP (s), and CPU + FPGA (one or more) can meet the performance requirements of real-time processing, but their power consumption is above 10 W, or even more than 100 W. A dedicated chip that fully implements the imaging algorithm can achieve better results in real-time, and low power consumption and is suitable for applications with strict power constraints, but the scheme hardens the algorithm, resulting in poor flexibility. ASIP (Application Specific Instruction Set Processor) is a dedicated processor solution between a general-purpose processor and an application specific integrated circuit (ASIC). This processor combines the flexibility of a general-purpose processor and the efficiency of an ASIC. In order to achieve a good trade-off between flexibility and processing efficiency, the development of a dedicated processor that is capable of fully implementing the SAR imaging process is an effective solution to meet its power consumption and real-time requirements for spaceborne/airborne SAR processing.

The chirp scaling algorithm (CSA) is one of the most commonly used algorithms for SAR imaging [11]. Its calculations mainly include Fast Fourier Transformation/Inverse Fast Fourier Transform (FFT/IFFT), phase multiplication, interpolation, etc., especially FFT/IFFT operations account for the highest proportion. The accuracy requirements and computing flow of these operations are different. Therefore, how to design an array structure and storage structure suitable for such processing is a key issue to be solved.

With the progress of integrated circuit (IC) technology, more processing units and memory blocks can be integrated on a single chip. Based on the abundant computational and memory resources on the chip, to make full use of bandwidth resources, this paper proposes a heterogeneous array structure that efficiently supports CSA imaging processing by combining block parallelism and pipeline processing while buffering the intermediate results on-chip.

It can support the parallel and pipeline processing and increases the maximum utilization of computing units. Moreover, we have designed an on-chip multi-level data buffer structure matching the heterogeneous array structure to ensure data supply for pipeline processing. This solution can reduce the complexity of the system while improving real-time performance. 

The paper is organized as follows. Section 2 outlines related work and background. Section 3 analyzes the characteristics of CSA and proposes the design of the processor. Section 4 presents the heterogeneous architecture implementations. We present the evaluation of experimental results in Section 5, and the conclusions in Section 6.

## 2. Related Work

Digital signal processors (DSPs), CPUs, and graphics processing units (GPUs) have respective advantages in real-time SAR processing. As the system adopts CPU, it has good flexibility and portability [12]. However, their power efficiency for computing is quite low, which is a bottleneck in real-time SAR applications. Due to GPU’s powerful parallel computation capability and programmability, the new method makes full use of GPU’s powerful computation ability, which effectively improves the real-time quality of SAR scene generation [13,14,15,16]. At present, the GPU + CPU method can effectively combine the advantages of the two processors to improve imaging efficiency [17,18]. However, the average power consumption which is up to 150 W, limits the application of GPU in micro air vehicles.

Nowadays, high capability DSPs easily realize many complex theories and algorithms on hardware, and promote the development of SAR technology [19,20,21]. In 2003, Hanover University implemented a SAR real-time processing system using a multi-DSP architecture. This system uses highly parallel digital signal processor technology (HiPAR-DSP) for SAR signal processing [22]. The Indian Space Research Organization (IRSO) developed the SAR Specialized Processor (NRTP) based on Analog Devices’ DSP multiprocessor, which approximates the real-time imaging of SAR [23]. However, for some applications with strictly constrained power, DSP has lower energy efficiency, resulting in lower imaging efficiency.

The rapid development of field-programmable gate array (FPGA) has been one of the most important technologies of realizing digital signal processing. With its rich on-chip memory and computational resources, FPGA can be configured as a SAR imaging platform to meet the high throughput rate SAR signal processing requirements [24,25,26]. An FPGA based on fault-tolerant architecture (Xilinx Virtex-II Pro) is applied to SAR processing systems [27,28]. In 2006, the University of Florida developed a high-performance heterogeneous spatial computing framework based on hardware/software interfaces. In this architecture, the CPU is responsible for scheduling and task management, and the FPGA acts as a coprocessor for computational acceleration [29]. With the rapid development of storage capacity and computing power of commercial FPGAs, SAR real-time imaging systems can all be built by FPGA (Xilinx Virtex-6) [30]. However, for highly complex algorithms, the development cycle of FPGA is relatively long.

For the real-time requirements and physical implementation limitations of SAR imaging, ASIC implementation is generally employed [31,32]. The Massachusetts Institute of Technology (MIT) Lincoln Laboratory uses bit-level systolic-array technology to design a SAR signal processor with high throughput and low power consumption [33]. The jet propulsion laboratory has also developed an airborne SAR processing system using a VLSI+SOC (very large scale integration+system on chip) hardware solution [10]. The processor’s low power consumption and small size make it suitable for small SAR imaging systems.

In general, the DSP solution is used to implement SAR imaging through software programming. Since the DSP is designed for general purposes, this implementation has high flexibility and a short design cycle. It is more suitable for real-time SAR imaging than a CPU, but for low power applications, it is still not the most suitable choice. The ASIC solution for SAR imaging has the optimal power and performance for a single computational process. However, SAR imaging is a combination of multiple calculations on one device, which causes the design cost and power consumption of SAR imaging to soar, the design cycle to become longer, and poor flexibility. ASIP makes a good trade-off between the high flexibility of a general purpose processor and the high processing efficiency of an ASIC, and can be tailored and optimized for a certain type of algorithm or domain application to meet constraints such as performance, area, and power consumption. Moreover, it can effectively reduce design cycles and the design risk. Thus, many advantages of ASIP make it a very important implementation method in the field of signal processing.

Making trade-offs between speed, cost, power consumption, and flexibility, ASIP design methodology in the design of SAR real-time signal processing system can not only satisfy the real-time and performance requirements of aerospace systems, but also shorten the lead time of the processors. ASIP, when designed with a specific architecture with higher parallelism and higher complexity, also has good scalability. Therefore, we have designed a dedicated processor that can fully implement the SAR imaging process to meet the power consumption and real-time requirements of the application environment.

## 3. Processor Architecture Design

The CSA is one of the most commonly used algorithms for SAR imaging [11]. Compared with other algorithms, the CSA has the advantages of a simple operation process, low computational complexity, and high imaging efficiency. On the other hand, the CSA improves the fidelity of the image, especially the preservation of the phase information. Moreover, the CSA can adapt to different radar scanning modes, for example, spotlight, strip-map, scan SAR, sliding spotlight, Tops, and Mosaic modes [34,35].

### 3.1. CSA Flow Analysis

**Figure 1:**
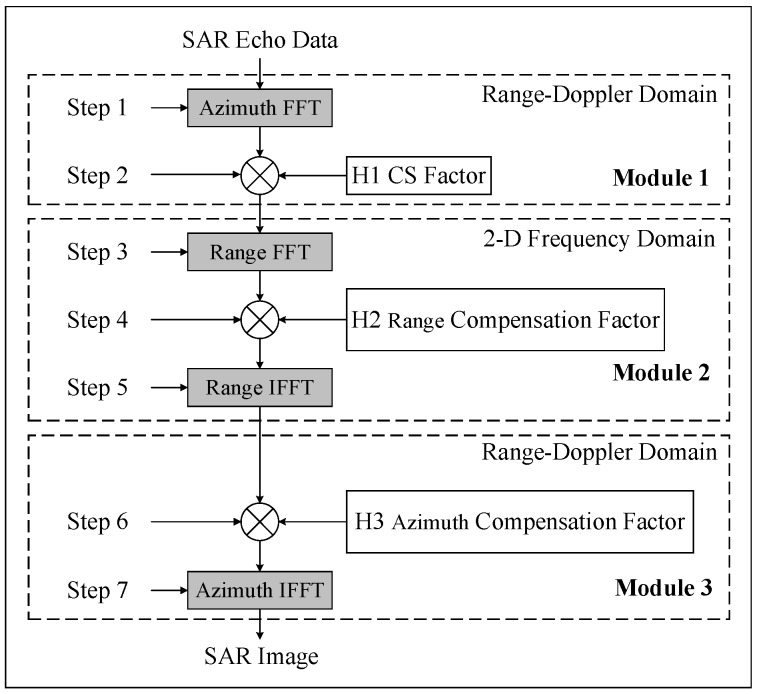
Chirp scaling algorithm (CSA) flow chart. SAR: synthetic aperture radar.

The imaging principle of the CSA is shown in Figure 1. The CSA can be divided into three modules according to functions, or divided into seven steps according to the operation sequence. The algorithm is executed step by step, and in the algorithm process, we perform the alternating operation of FFT/IFFT and phase compensation. To perform a SAR imaging, four Fourier transform and three-phase multiplication are needed. 

The Q-point FFT/IFFT can be decomposed into $2Q\log_{2}Q$ real multiplications and $3Q\log_{2}Q$ real additions [36]. Table 1 lists the computation quantity of the seven-step operation.

From Table 1, we can see that the proportion of FFT(IFFT) in all operations is:

(1) $$W\;=\;\frac{\left(2+2+3+3\right)NM\log_{2}M\;+\left(2+2+3+3\right)NM\log_{2}N}{10NM\log_{2}M\;+\;10NM\log_{2}N\;+18NM\;}$$

For different imaging matrix sizes, the proportion W of FFT(IFFT) is slightly different, as shown in Table 2. It can be shown from Table 2 that the W values are basically above 90% and can reach up to 95% as the matrix size becomes larger. Therefore, accelerating the FFT/IFFT operation will inevitably reduce the imaging time and optimize the imaging efficiency.

### 3.2. Computation Flow Strategy

**Figure 2:**
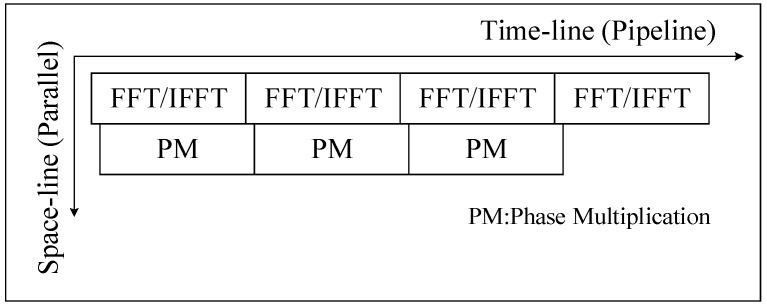
SAR imaging flow.

In the imaging process, we take the block imaging method and perform parallel processing between blocks. In the algorithm process, four FFT/IFFT and three phase operations are pipelined according to the algorithm flow, while each multi-range (multi-azimuth) FFT/IFFT and phase operation can be parallel processing individually. To organize the pipeline processing of two types of operations in SAR imaging, we designed a calculation process based on space–time flow (ST-Flow), as shown in Figure 2. At a time, in space, multi-line FFT/IFFT can be performed in parallel, and phase compensation operation can be calculated simultaneously at multiple points, so no calculation unit is idle. On the timeline, data is continuously fed into the processing unit, and the calculation unit does not have a stall due to waiting for data. With this ST-Flow, SAR imaging can be done in a continuous process.

### 3.3. Heterogeneous Arrays

CSA includes scalar operation for phase multiplication and vector operation FFT (IFFT). As Table 2 shown, FFT/IFFT operations account for up to 95% of SAR imaging, so accelerating the FFT/IFFT operation efficiently is the most important approach for imaging processors.

The fixed-point FFT/IFFT operation with lower accuracy has a small loss of imaging accuracy, and can significantly improve the processing throughput. In [37], the quantization error power of the fixed-point processing CSA was evaluated in detail. The analysis results showed that as the word length increases from 12 to 16, the quantization error power remains essentially unchanged, and the imaging quality with a 15 or 16-bit word length is very close to that of a single precision floating-point. Therefore, we design PE arrays to support 12-bit, 14-bit, and 16-bit fixed-point FFT/IFFT. For applications with lower accuracy requirements, low-bit width operation can be selected. 

However, the phase compensation operation requires high precision and must use floating-point arithmetic operations. Based on the earlier description and discussion, a heterogeneous array is designed, which includes two types of computing units named PE and FPE. PE is used for FFT/IFFT operation and FPE is used for phase compensation operation.

**Figure 3:**
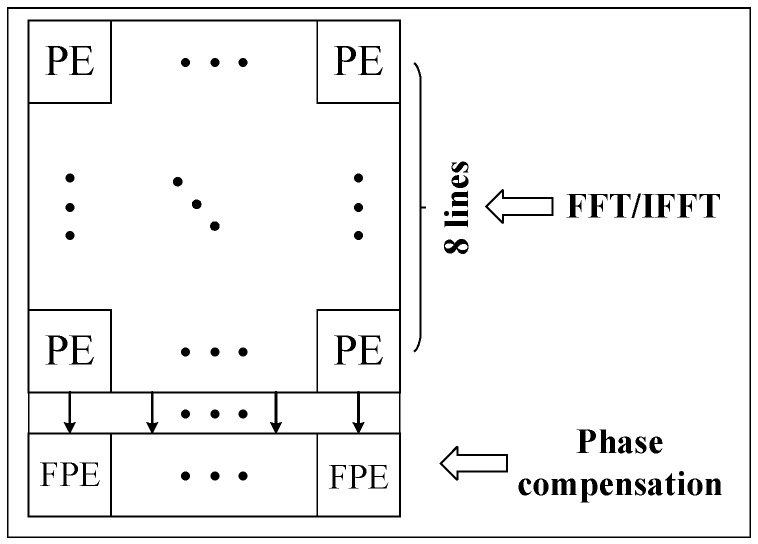
PE array structure diagram. PE: computing unit for FFT/IFFT operation in a heterogenous array.

Since the operation ratio of FFT/IFFT against phase compensation is approximately 9:1, the configuration of PE and FPE should also follow this proportional relationship. For smaller matrix sizes, the ratio is near 90%; to meet the different matrix sizes, we design the processing array, in which the ratio of PE and FPE is 8:1, as shown in Figure 3.

In CSA flow, each range/azimuth FFT/IFFT operation is relatively independent, and there is no data dependency between range/azimuth, so each range/azimuth FFT/IFFT operation can be performed in parallel. Moreover, in the FFT/IFFT operation, each butterfly operation is relatively independent, and multiple butterfly operations can be performed in parallel. The phase compensation process performs independent operations at a single point so that multiple independent operations can be performed in parallel. 

**Figure 4:**
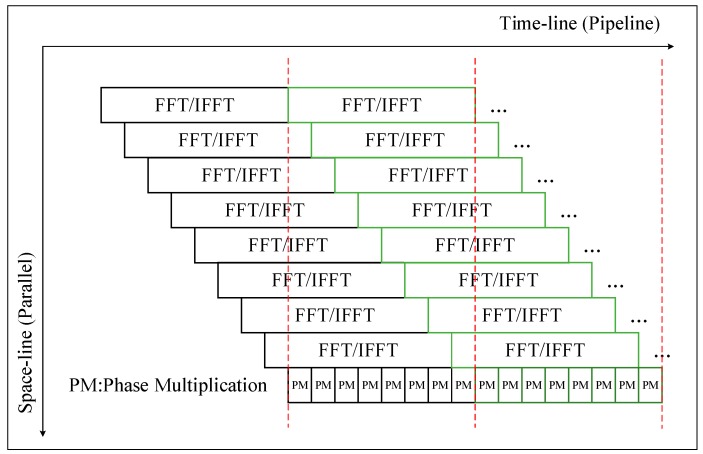
Pipeline between FFT/IFFT and phase operation.

In CSA flow, four FFT/IFFT and three phase operations are data dependent; they are processed in the pipeline. As shown in Figure 4, to establish a pipeline between the FFT and the phase operation, the parallel FFT/IFFT differ by 1/8 computation cycles.

### 3.4. Data Placement and Simultaneous Access

**Figure 5:**
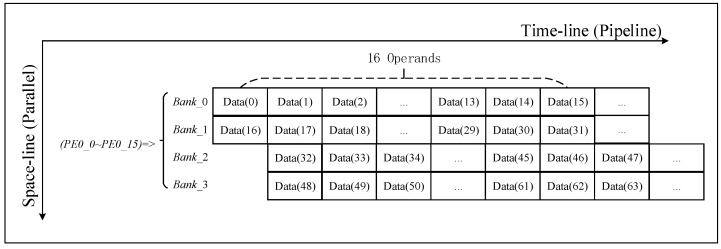
16 PEs perform base-4 butterfly operation timing diagram for four banks.

In the FFT/IFFT process, the data transfer has a bit-reverse address sequence. To support this data access pattern, we use a multi-bank distributed data placement strategy, as shown in Table 3. According to the calculation requirements, one row of PE parallel performs 16 butterfly operations, and needs to provide 32 data at the same time. Therefore, data access is performed in parallel. As shown in Figure 5, 32 data are simultaneously accessed from Bank 0 and Bank 1 in the first cycle. In the second cycle, data are read simultaneously from Bank 2 and Bank 3. Bank selection and the address in a bank are generated to follow each step in the FFT/IFFT processing flow. Although each PE performs a different FFT/IFFT operation, they use similar data placement and access strategies. 

**Figure 6:**
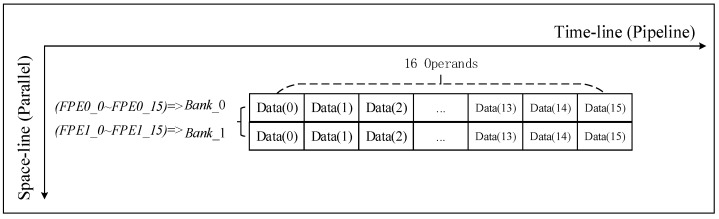
32 FPEs perform phase operation for two banks. FPE: computing unit in a heterogeneous array used for phase compensation operation.

There is no special requirement for the sequence of data in the phase compensation calculation process; therefore, as shown in Figure 6, the calculation process only needs to access the data in parallel.

## 4. Architectural Implementations

### 4.1. Overall Architecture

**Figure 7:**
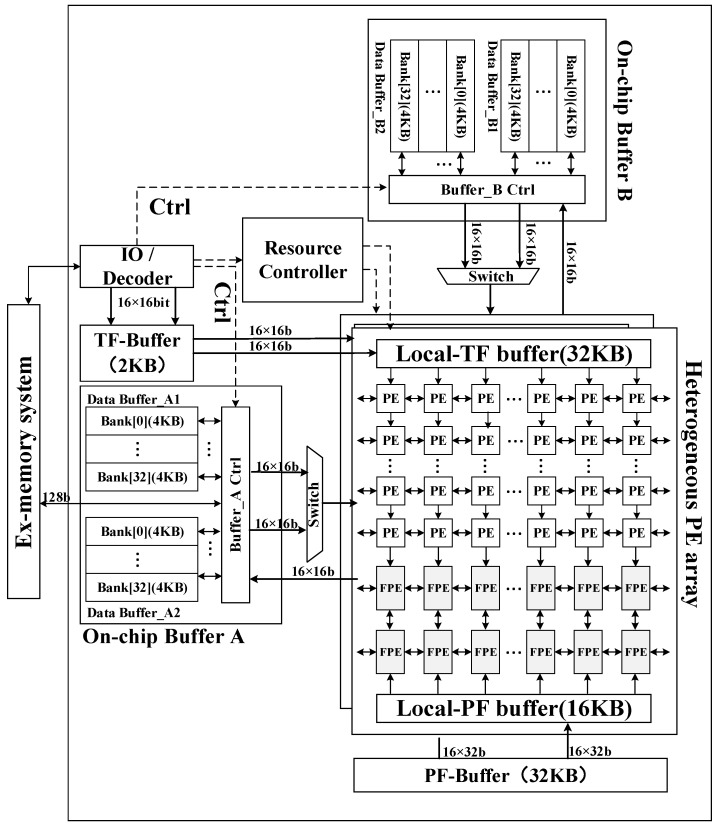
Top-level architecture of processor.

A highly efficient heterogeneous processor for SAR imaging is designed. Figure 7 shows the top-level architecture of the proposed SAR imaging processor. This section describes the overall hardware block diagram and functional modules. Essentially, the architecture consists of three major components: a hybrid–PE array, an on-chip buffer module, and a data systolic engine.

To meet the throughput requirement of SAR imaging, two identical sets of heterogeneous arrays are implemented, which can perform different block imaging processing computations in parallel. Each of the heterogeneous arrays contains 16 × 16 PEs and 2 × 16 FPEs. The number ratio of PE and FPE satisfies the proportional relationship of 8:1.

To feed the processing array with adequate data supply, three types of buffers are implemented on chip. In a processing array, all the data banks for 16-line PEs and two-line FPEs are organized as a 264-KB data buffer with two sub-buffers, each of which contains 32 banks for PEs and one bank for an FPE. A 32-KB twiddle factor dedicated local buffer (Local-TF buffer) and a 16-KB phase factor dedicated local buffer (Local-PF buffer) for the phase compensation operation is also implemented inside a processing array.

To organize the data transfer between off-chip RAM and on-chip buffers, a data systolic engine is implemented. With this data systolic engine, the input raw image echo can be read and the imaging output can be written back following the processing flow.

### 4.2. Heterogeneous PE Arrays

Each PE pipelined performs a four-point butterfly operation in six cycles, and all of the PE in a row parallel perform butterfly operations in a block. During the FFT/IFFT operation, all 64 input data are sent to one row of PEs in two cycles from the data buffer, and the 64 output data are written back to the data buffer in two cycles.

In a heterogeneous array, as shown in Figure 7, PEs are interconnected to pass a twiddle factor, the Local-TF buffer distributes the twiddle factor to the PE from top to bottom. The twiddle factor passes two rows down each cycle, and the required twiddle factors are assigned to 16 rows of PEs in eight cycles. Besides, each PE supports zero-padding to expand the raw data to an integer power of two.

During the phase compensation operation, the two input data banks send 32 input data to two rows of FPEs (32 FPEs) in parallel. The Local-PF buffer passes and distributes the phase compensation factor from bottom to top.

### 4.3. Alternate Systolic-Memory and On-Chip Buffer Organization

**Figure 8:**
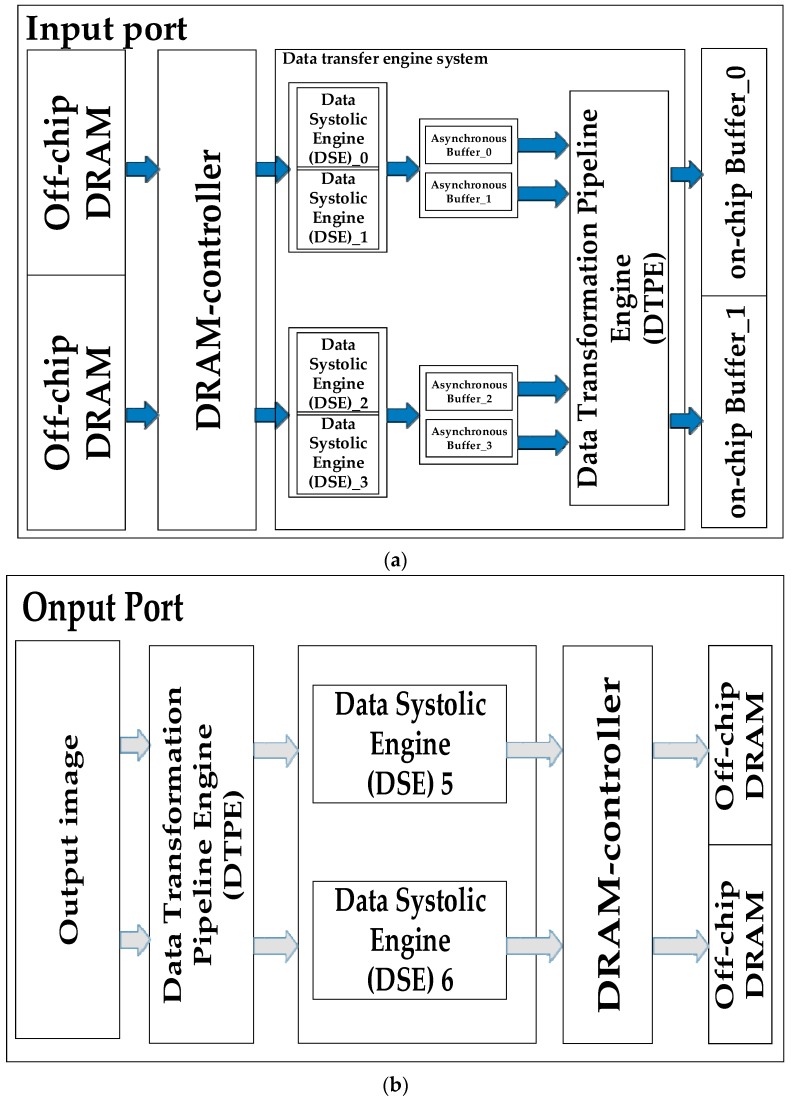
Memory hierarchy architecture. (**a**): Input Port; (**b**): Output Port.

Since on-chip memory space is limited, all of the radar echo data is stored in the external memory first. As shown in Figure 8, the data systolic engine (DSE) fetches the data from dynamic random access memory (DRAM) and pushes the data into on-chip memory. To hide the communication latency of data transfer between DSEs and arithmetic components, we employ the alternate systolic technique. In order to avoid DSE competition in hardware resources, we use two alternate systolic memory modules for each of the input/output interfaces for the whole system. At the same time, we adopt two DSE channels for input data and weight at the input end. The proposed memory architecture can provide 4 GB/s of read/write memory bandwidth at 250-MHz frequency to satisfy the data requirements of the processor.

As shown in Figure 8, our storage architecture consists of three layers: DRAM, a data transfer engine system, and an on-chip buffer. Since the on-chip storage resources are limited in size, all the pending radar echo data is first stored in off-chip memory (DRAM). During data processing, the data is first cached by the data transfer engine system into the on-chip buffer, and then sent to the PE array for processing by the on-chip buffer. As shown in Figure 8, in order to hide the communication latency between the off-chip memory and the on-chip buffer, we use the double-buffered data alternate transmission method.

### 4.4. Resource Controller

The resource controller is responsible for allocating the execution unit and arranging the access flow of the on-chip buffer.

Two imaging blocks are respectively assigned to two arrays for parallel processing. The FFT/IFFT and phase compensation operations are involved in the intra-block processing, so the PE is assigned to the FFT/IFFT during the calculation and the FPE is assigned to the phase compensation operation.

According to the designed data mapping and access strategy, in order to support the parallel access of data, the resource controller allocates bank and bank addresses for each range of data. When performing range FFT/IFFT, each row of data is stored in four banks according to a distributed storage strategy.

**Figure 9:**
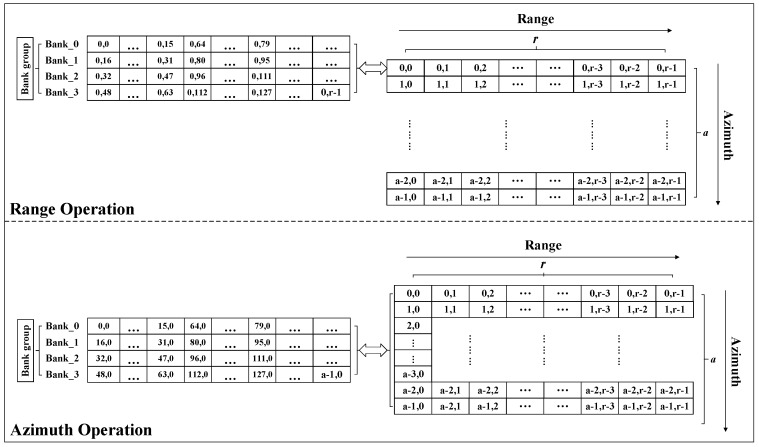
Data access pattern.

As shown in Figure 9, we take a row of 1024 points as an example (*r* = 1024). When performing FFT/IFFT, 1024 points are segmented and stored in four banks according to the distributed storage strategy. A total of 16 consecutive points are used as a segment, in which approximately 0 to 15 are placed in Bank_0, 16 to 31 are placed in Bank_1, 32 to 47 are placed in Bank_2, and 48 to 63 are placed in Bank_3; the above operation is repeated until all data of 256 segments are stored. A base-4 FFT/IFFT operation at 1024 points requires a total of five levels of operation. The calculation process uses multi-bank parallel data access. Taking the first stage as an example, data 0 to 31 is read from Bank_0 and Bank_1 in the first cycle, and data 992 to 1023 is read from Bank_2 and Bank_3 in the second cycle. The latter four levels of the operational data access process are similar to the first level.

Similarly, when performing azimuth FFT/IFFT, each azimuth of data is stored in four banks according to the storage strategy (taking 1024 points as an example, a = 1024). The data access process is similar to the FFT/IFFT range.

**Figure 10:**
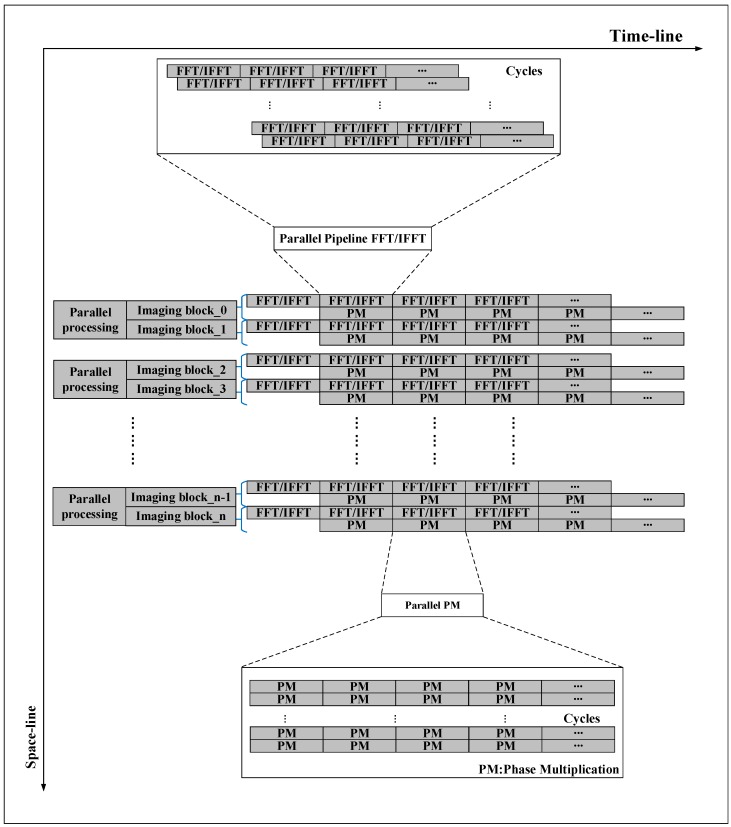
Space–time flow (ST-Flow) of imaging processing.

SAR imaging is a continuous process with huge differences in operational density between FFT/IFFT and the phase compensation operation. For the characteristics of the computational process, we have designed a way to organize the processing of SAR imaging in space and time flow (ST-Flow), as shown in Figure 10.

Taking 1024 points FFT/IFFT as an example, each FPE performs a one-point phase compensation operation in one cycle, and all the FPE in a row parallel perform phase compensation operations. During the phase compensation operation, all 16-input data are sent to one row of FPEs in one cycle from the data buffer, and 16 output data are written back to the data buffer in one cycle. It can be seen that the 1024-point phase compensation operation requires 64 cycles. In order to satisfy the task saturation and parallelism of the parallel pipeline between phase compensation and FFT/IFFT, the resource controller sets the start time for each row of PE to be delayed by 64 cycles from the previous row. Considering the different matrix sizes, the ratio of PEs to FPEs is configured to be 8:1, so for larger matrices, the FPE will be idle. During the processing of the FPE, it is necessary to wait for the PE to complete the FFT operation before starting the processing of the next frame.

## 5. Processor Performance Evaluation

**Figure 11:**
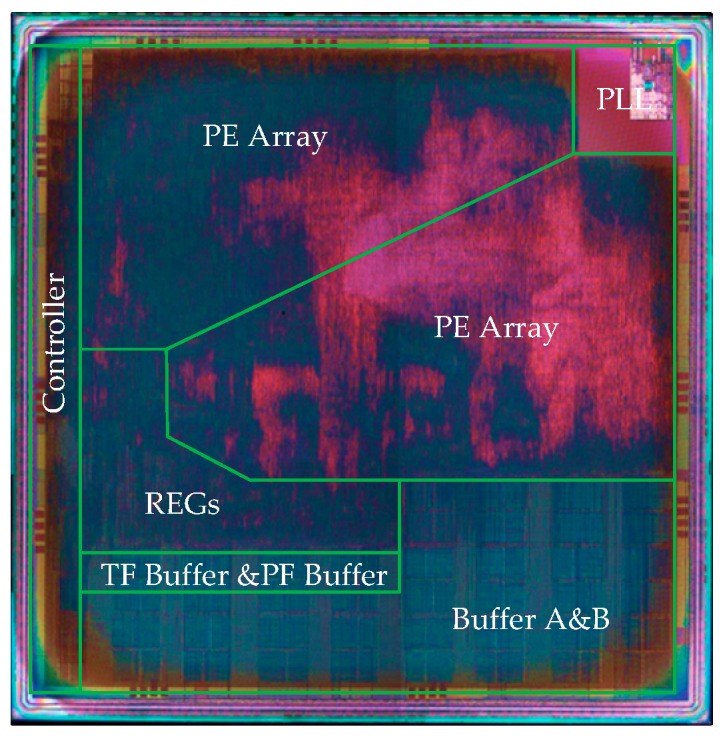
Die photograph of the chip.

We implemented the SAR imaging processor at 65-nm CMOS (complementary metal oxide semiconductor) technology with 1.2 V of supply voltage using Synopsys tools. Figure 11 shows the die photograph of the chip. In the evaluation, the CS imaging algorithm is selected as the benchmark. 

### 5.1. Performance Analysis

In this section, we configure the processor with fixed-point PE and single-precision floating-point FPE. We evaluate the processor performance at 200 MHz with different fixed-point lengths. The test echo data matrix size is 16,384 × 16,384. We perform two operations in parallel on the heterogeneous PE, which can take advantage of the computing power and increase the throughput. When the CSA is processed in heterogeneous PE mode, the throughput is achieved to 115.2 Giga operations per second (GOPS), with 463 mW of power consumption. As shown in Table 4, when all the imaging processes use single-precision floating-point units, the power consumption of the processor is up to 713 mW, and its energy efficiency is only 67% of the fixed/floating point heterogeneous imaging mode. Also, the processor can reduce a small amount of power consumption when selecting low-bit fixed-point FFT/IFFT operation. The processor consumes 463 mW for 16-bit fixed-point FFT/IFFT and reduces to 454 mW for 12-bit fixed-point FFT/IFFT, as shown in Table 4.

### 5.2. Array Utilization Analysis

As shown in Table 5, we can see that in the algorithm processing, the ST-flow two-dimensional parallel pipeline achieves better array utilization than one-dimensional time-based computational flow (TI-flow). The high utilization of the array can increase the throughput of the system. The time-based computational flow (TI-flow) that is employed in existing processors is inefficient for SAR imaging processing. As shown in Table 5, in the ST-Flow mode, the FFT operation and the phase mean (PM) operation are pipelined, the throughput reaches 115.2 GOPS, the resource utilization rate can reach 98.8%, and the energy efficiency is 0.24 GOP/mW. In TI-Flow mode, the FFT operation and the PM operation are executed sequentially, the throughput is only 62.6 GOPS, the resource utilization rate is 54.3%, and the energy efficiency is only 0.16 GOP/mW. Compared with the TI-Flow mode, the resource utilization in ST-Flow mode significantly increases, the throughput increases by 84.5%, and the average power consumption only increases by 21.2%.

### 5.3. Analyzes of Array Scalability

**Figure 12:**
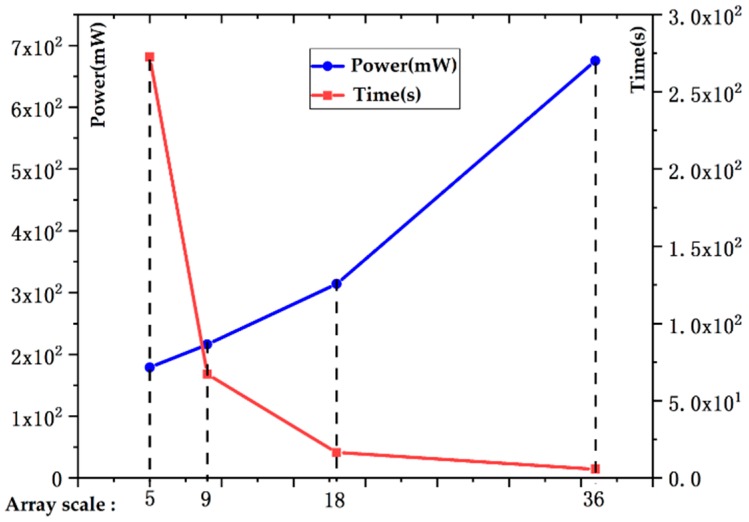
Power consumption and imaging time with different array sizes.

**Figure 13:**
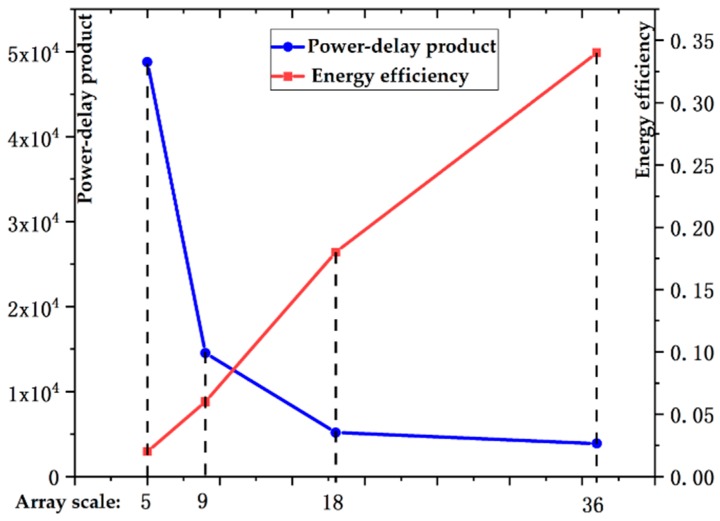
Energy efficiency and power-delay product with different array sizes.

We analyze the performance of a single heterogeneous array, as shown in Figure 12 and Figure 13. On the horizontal (*X*) axis, the numbers 5, 9, 18, and 36 represent the array scales of 5 × 4, 9 × 8, 18 × 16, and 36 × 32, respectively.

As the size of the array increases, the throughput and imaging efficiency of the system increase significantly, but the power consumption of the processor also rises sharply. In general, the power-delay product and energy efficiency of large PE arrays are better than those of small PE arrays. On the other hand, the array size must be closely matched to the buffer size; an oversized or undersized array configuration will result in wasted PE resources or low memory bandwidth utilization. Therefore, the size of a single heterogeneous processing array is designed to be 18 × 16 after a trade-off between the chip implementation complexity and processing performance.

### 5.4. Comparison with Other Schemes

Table 6 lists the SAR imaging time for different sizes of input. For the ordinary SAR radar (for instance, the Chinese Gaofen-3 satellite, pulse repetition frequency: 2000 Hz), the real-time processing time of 16,384 × 16,384 SAR raw data requires 8 s. The proposed scheme can meet the real-time requirements.

The power consumption and SAR imaging time for other studies are also listed in Table 6. As can be seen from Table 6, the power consumption of the proposed scheme is the smallest, because the proposed scheme can completely realize the entire SAR imaging process without additional microcontroller unit (MCU) or CPU. Similar to [15], the Mobile-GPU architecture uses a lower power cost (5 W) to achieve better real-time performance. Compared with [15], the proposed architecture is better in performance-to-power ratio and improves by a factor of 230.4. From the real-time performance perspective, the CPU + GPU scheme is the best, but its power consumption exceeds 300 W. The real-time performance of the proposed scheme is only 8.6% of [17], but the performance-to-power ratio improves by a factor of 63.4. Table 7 shows the comparison of the proposed scheme and related research in real-time performance. As can be seen from Table 7, compared with [15], the speedup ratio reached 21.33.

### 5.5. SAR Imaging Quality Evaluation

**Figure 14:**
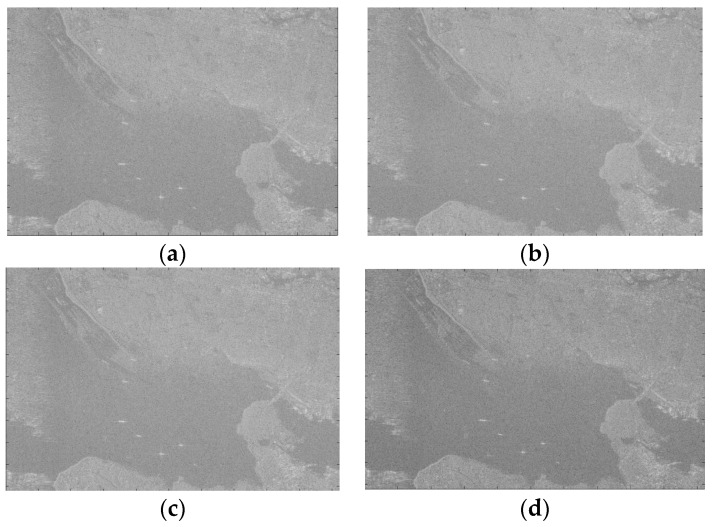
The scene SAR imaging results for different fixed-point length FFT. (**a**) 12-bit fixed-point FFT; (**b**) 14-bit fixed-point FFT; (**c**) 16-bit fixed-point FFT; (**d**) single-precision float-point FFT.

We compared the scene SAR imaging results of different fixed-point length FFT. Radar data were obtained from RADARSAT-1 of Canada (width: 50 km; resolution: 6 m) [38]. The imaging effect is shown in Figure 14. 

For the actual scenes, the mean square error (*MSE*), peak signal-to-noise ratio (*PSNR*) [39], structural similarity index (*SSIM*) [40], and radiometric resolution (*RL*) [41] are commonly adopted to evaluate SAR imaging quality. 

Sufficient imaging accuracy can be achieved with single-precision floating-point imaging. Fixed-point processing methods will cause a certain loss of precision. We take the single-precision floating-point imaging as the test reference to evaluate the fixed-point FFT SAR image quality. 

The *MSE* is adopted to calculate the squared intensity difference between the pixels of the partial fixed-point image and the pixels of the full single-precision floating-point image. The *PSNR* is essentially the same as the *MSE*, but it is associated with the quantized gray level of the SAR image. The *MSE* and *PSNR* are calculated as shown in Formulas (2) and (3):

(2) $$MSE=\frac{1}{M\times N}\overset{M}{\underset{i=1}{\sum }}\overset{N}{\underset{j=1}{\sum }}{\left(f^{\prime }\left(i,j\right)-f\left(i,j\right)\right)}^{2}$$

(3) $$\;PSNR=10\log_{10}\frac{Q^{2}\times M\times N}{\sum_{i=1}^{M}\sum_{j=1}^{N}{\left(f^{\prime }\left(i,j\right)-f\left(i,j\right)\right)}^{2}}\;$$

where $f^{\prime }\left(i,j\right)$ and f(i,j) represent the image pixels to be evaluated and the reference image pixels, respectively; *M*, *N* represent the length and width of the image, respectively. *Q* represents the gray level of the image (*Q* = 255).

*PSNR* and *MSE* are simple and straightforward SAR image quality assessments based on the visibility of errors. Due to the *PSNR* index not being exactly the same as the visual quality seen by the human eye, the evaluation requirements of the human visual system (HVS) cannot be met [40]. Therefore, we also adopt *SSIM* (the Structural Similarity Index) to evaluate the SAR images. As shown in Formula (4):

(4) $$SSIM\left(x,y\right)=\frac{\left(2\varphi_{x}\varphi_{y}+\varepsilon_{1}\right)\left(2\delta_{xy}+\varepsilon_{2}\right)}{\left(\varphi_{x}^{2}+\varphi_{y}^{2}+\varepsilon_{1}\right)\left(\delta_{x}^{2}+\delta_{y}^{2}+\varepsilon_{2}\right)}$$

where $\delta_{x}^{2}$ represents the fixed-point image variance, and $\delta_{y}^{2}$ represents the single-precision floating-point image variance; $\varphi_{x}$ represents the mean value of the fixed-point image, and $\varphi_{y}$ represents the mean value of the single-precision floating-point image. The *SSIM* value range is [0, 1], and the larger the SSIM value, the smaller image distortion. 

*RL* is also a very important evaluation indicator. *RL* is adopted to evaluate the minimum variation of target reflection that radar sensors can distinguish. As shown in Formula (5):

(5) $$RL=10\;\log_{10}\left(\frac{\alpha }{\beta }+1\right)$$

where *α* represents the standard deviation of the image, and *β* represents the mean value of the image.

Table 8 lists the loss of precision due to the different data widths. As can be seen from Table 8, the *PSNR* value of a partial 16-bit fixed-point image can reach 29.1 dB, the results show that the partial 16-bit fixed-point image and the single-precision floating-point image differ only by 0.02 and 0.05 dB on the two indexes of *SSIM* and *RL*, respectively. For the actual scene SAR imaging, compared with a single-precision floating-point image, the accuracy loss of a partial 16-bit fixed-point image is within 2%.

Phase is also important information for a SAR image. The phase mean (*PM*) and phase deviations (*PD*) are estimated by the method proposed in [42]. Table 9 lists the phase precision with different fixed-point SAR imaging. As can be seen from Table 9, the loss of phase precision with partial 16-bit fixed-point imaging is less than 3%.

**Figure 15:**
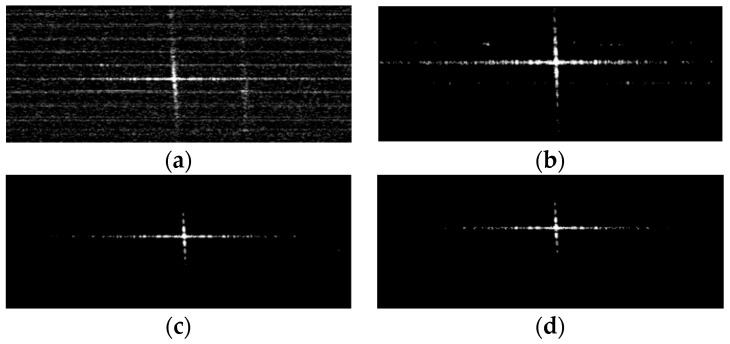
The point target SAR imaging results for different fixed-point length FFT. (**a**) 12-bit fixed-point FFT; (**b**) 14-bit fixed-point FFT; (**c**) 16-bit fixed-point FFT; (**d**) single-precision float-point FFT.

For the point target imaging quality evaluation, we adopted the point target simulation echo data. We compared the point target SAR imaging results for FFT with different fixed-point lengths, as shown in Figure 15. For the point target image, spatial resolution (RES), peak side lobe ratio (PSLR) and integrated side lobe ratio (ISLR) are commonly adopted to assess imaging quality [38,43]. Table 10 shows the results of the point targets imaging quality assessment and comparison.

For partial 16-bit fixed-point imaging, in the azimuth direction, the PSLR and ISLR precision loss of the image are 0.3% and 0.8%, respectively; the RES precision loss is 0.2%. In the range direction, the PSLR and ISLR precision losses of the image are 0.2% and 0.2%, respectively; the RES precision loss is 0.7%.

According to the actual scene and the point target image quantization analysis, as shown in Table 8, Table 9 and Table 10, the partial 16-bit fixed-point imaging accuracy is close to the single-precision floating-point imaging accuracy, which meets the requirements of on-orbit SAR imaging applications.

## 6. Conclusions

This paper proposes a heterogeneous imaging processor using fixed-floating point heterogeneous parallel acceleration technology to perform SAR imaging in the aerospace field. The processor consists of two 18 × 16 heterogeneous arrays that provide 115.2 GOPS throughput. To improve energy efficiency, each array supports fixed-floating hybrid calculations to take full advantage of computing resources, which can increase the throughput of imaging processing by 1.82 times. At the same time, the PE array can be partitioned by rows through a sensible algorithm-to-hardware architecture mapping, process the imaging process in parallel, provide high-utilization hardware resources, and improve the efficiency by a factor of 1.5. A single processor requires 8 s and consumes 463 mW to process SAR raw data with a granularity of 16,384 × 16,384, which meets the limits real-time and power consumption of the on-orbit SAR imaging platform. The proposed solution also has good scalability, by extending the size of the processor array, the real-time requirements of larger-scale SAR imaging can be met.

## Figures and Tables

**Table 1:** Computational statistics of CSA.

Calculation Content	Step	Real-Multi	Real-Add	Total
**Azimuth-FFT ^1^**	1	$\;2NM\log_{2}M\;$ ^2^	$\;3NM\log_{2}M\;$	$\;5NM\log_{2}M\;$
**CS Factor-Multi ^3^**	2	4NM	2NM	6NM
**Range-FFT**	3	$\;2NM\log_{2}N\;$	$\;3NM\log_{2}N\;$	$\;5NM\log_{2}N\;$
**RC Factor ^4^-Multi**	4	4 *NM*	2NM	6NM
**Range-IFFT ^5^**	5	$\;2NM\log_{2}N\;$	$\;3NM\log_{2}N\;$	$\;5NM\log_{2}N\;$
**AC Factor ^6^-Multi**	6	4NM	2NM	6NM
**Azimuth-IFFT**	7	$\;2NM\log_{2}M\;$	$\;3NM\log_{2}M\;$	$\;5NM\log_{2}M\;$
**Total**	-	$\;4NM\log_{2}M$ + 4*NM*$\log_{2}N\;$ + 12NM	$\;6NM\log_{2}M\;$ + $6NM\log_{2}N\;$ + 6NM	$\;10NM\log_{2}M$ + $10NM\log_{2}N$ + 18NM

^1^**FFT**: Fast Fourier Transformation; ^2^*M*: Azimuth direction sample numbers; *N*: Range direction sample numbers; ^3^ CS Factor: Chirp Scaling Factor; ^4^ RC Factor: Range Compensation Factor; ^5^ IFFT: Inverse Fast Fourier Transform. ^6^ AC Factor: Azimuth Compensation Factor.

**Table 2:** Computational load statistics.

Image Size	256 × 256	1024 × 1024	4096 × 4096	16,384 × 16,384	65,536 × 65,536
**FFT Computational Load**	$\;10^{7}\;$	$\;2.1\times 10^{8}\;$	$\;4.1\times 10^{9}\;$	$\;7.6\times 10^{10}\;$	$\;1.2\times 10^{12}\;$
**Phase Compensation Computational Load**	$\;10^{6}\;$	$\;1.8\times 10^{7}\;$	$\;3\times 10^{8}\;$	$\;4.8\times 10^{9}\;$	$\;1.2\times 10^{10}\;$
***W*-Value**	89.8%	91.7%	93%	94%	94.7%

**Table 3:** 4096 points input data storage in four banks.

Bank_NO.	Input Data Storage Status in Bank			
Bank_0	0–15	64–79	-	-
Bank_1	16–31	80–95	-	-
Bank_2	32–47	96–111	-	-
Bank_3	48–63	112–127	-	4080–4095

**Table 4:** System performance assessment with different fixed-point length FFT.^1^

PE Bit-Width (Bits)	12	14	16	Single-Precision Floating
Throughput (GOPS)	115.2	115.2	115.2	115.2
Power (mW)	454	459	463	713
Energy efficiency (GOPS/mW)	0.254	0.250	0.240	0.16

^1^Table 4 provides statistics on throughput, power consumption, and energy efficiency for the entire heterogeneous processor.

**Table 5:** Array utilization with ST-flow and time-based computational flow (TI-flow). GOPS: Giga operations per second.

-	ST-Flow	TI-Flow		
		FFT	Phase Compensation	Overall
Array utilizations	98.8%	88.9%	11.1%	54.3%
Throughput (GOPS)	115.2	102.4	12.8	62.6
Power (mW)	463	435	317	382
Energy efficiency (GOP/mW)	0.24	-	-	0.16

**Table 6:** Comparison with previous works.

Architectural Model	Operating Frequency	Power Consumption	SAR Imaging Algorithms	Frame Size	SAR Signal Processing Time (s)
Proposed solution	200 MHZ	463 mW	CS	1024 × 1024	0.04
				2048 × 2048	0.15
				6472 × 3328	0.68
				16,384 × 16,384	8.2
				30,000 × 6000	5.54
				32,768 × 32,768	32.9
GPGPU [14]	-	>500 W	Omega-k	30,000 × 6000	8.5
CPU + GPU [18]	-	345 W	CS	32,768 ×32,768	2.8
Mobile-GPU [16]	2.3 GHZ	5 W	CS	2048 × 2048	3.2
Microprocessor + FPGA [15]	-	68 W	CS	6472 × 3328	8
CPU + ASIC [28]	100 MHZ	10 W	-	1024 × 1024	-

**Table 7:** Speed-up ratio to previous works.

Architectural	Imaging Time	Imaging Time in Proposed Solution	Speed-Up Ratio	Frame Size
CPU + ASIC [28]	-	0.04 s	-	1024 × 1024
Mobile-GPU [16]	3.2 s	0.15 s	21.33	2048 × 2048
Microprocessor + FPGA [15]	8 s	0.68 s	11.76	6472 × 3328
GPGPU [14]	8.5 s	5.54 s	1.54	30,000 × 6000
CPU + GPU [18]	2.8 s	32.9 s	0.086	32,768 × 32,768

**Table 8:** Quantitative evaluation of actual scene SAR imaging.

FFT Pro-Acc ^1^	*PSNR*^2^ (dB)	*MSE*^3^ (dB)	*SSIM*^4^ (dB)	*RL*^5^ (dB)
Single-precision float-point	∞	0	1	4.99
12-bit fixed-point	13.7	2765.2	0.23	4.11
14-bit fixed-point	22.4	377.4	0.77	4.71
16-bit fixed-point	29.1	81.8	0.98	4.94

^1^ FFT pro-acc: FFT processing accuracy; ^2^ PSNR: peak signal-to-noise ratio; ^3^ MSE: mean square error; ^4^ SSIM: Structural Similarity Index; ^5^ RL: Radiometric Resolution.

**Table 9:** Phase information evaluation of actual scene SAR imaging.

FFT Pro-Acc ^1^	*PM* ^2^	*PD* ^3^
Single-precision float-point	0.00244°	3.3026°
12-bit fixed-point	0.00916°	3.3054°
14-bit fixed-point	0.00398°	3.3032°
16-bit fixed-point	0.00252°	3.3026°

^1^ FFT pro-acc: FFT processing accuracy; ^2^ PM: phase mean; ^3^ PD: phase deviation.

**Table 10:** Quantitative evaluation of point target SAR imaging.

FFT Pro-Acc ^1^	Azimuth Direction			Range Direction		
	RES ^2^ (m)	PSLR ^3^ (dB)	ISLR ^4^ (dB)	RES (m)	PSLR (dB)	ISLR (dB)
Single-precision float-point	4.74	−12.91	−9.64	2.58	−13.31	−9.96
12-bit fixed-point	5.43	−5.68	−2.99	3.71	−5.88	−3.22
14-bit fixed-point	4.81	−11.85	−8.22	2.83	−11.55	−9.09
16-bit fixed-point	4.77	−12.86	−9.53	2.61(m)	−13.28	−9.93

^1^ FFT pro-acc: FFT processing accuracy; ^2^ RES: spatial resolution; ^3^ PSLR: peak side lobe ratio; ^4^ ISLR: integrated side lobe ratio.
